# Expert cognition in the production sequence of Acheulian cleavers at Gesher Benot Ya'aqov, Israel: A lithic and cognitive analysis

**DOI:** 10.1371/journal.pone.0188337

**Published:** 2017-11-16

**Authors:** Gadi Herzlinger, Thomas Wynn, Naama Goren-Inbar

**Affiliations:** 1 Institute of Archaeology, The Hebrew University of Jerusalem, Jerusalem, Israel; 2 The Jack, Joseph and Morton Mandel School for Advanced Studies in the Humanities, The Hebrew University of Jerusalem, Jerusalem, Israel; 3 Department of Anthropology, University of Colorado, Colorado Springs, Colorado, United States of America; Max Planck Institute for the Science of Human History, GERMANY

## Abstract

Stone cleavers are one of the most distinctive components of the Acheulian toolkit. These tools were produced as part of a long and complex reduction sequence and they provide indications for planning and remarkable knapping skill. These aspects hold implications regarding the cognitive complexity and abilities of their makers and users. In this study we have analyzed a cleaver assemblage originating from the Acheulian site of Gesher Benot Ya‘aqov, Israel, to provide a reconstruction of the *chaîne opératoire* which structured their production. This reduction sequence was taken as the basis for a cognitive analysis which allowed us to draw conclusion regarding numerous behavioral and cognitive aspects of the GBY hominins. The results indicate that the cleavers production incorporated a highly specific sequence of decisions and actions which resulted in three distinct modes of cleavers modification. Furthermore, the decision to produce a cleaver must have been taken very early in the sequence, thus differentiating its production from that of handaxes. The substantial predetermination and the specific reduction sequence provide evidence that the Gesher Benot Ya‘aqov hominins had a number of cognitive categories such as a general ‘tool concept’ and a more specific ‘cleaver concept’, setting them apart from earlier tool-producing hominins and extant tool-using non-human primates. Furthermore, it appears that the Gesher Benot Ya‘aqov lithic technology was governed by expert cognition, which is the kind of thinking typical of modern human experts in their various domains. Thus, the results provide direct indications that important components of modern cognition have been well established in the minds of the Gesher Benot Ya‘aqov hominins.

## Introduction

Cleavers are a morpho-techno-typological group within the wider family of bifacial tools, and are one of the most distinctive components of the Acheulian Technocomplex. They are defined on the basis of strict morphological and technological criteria of the blank on which it was modified and the morphology and design of the distal end, which is considered to be the working edge. For a tool to be classified as a cleaver it must be made on a flake blank and have a working edge unmodified by retouch [1]. The working edge must be straight or curved in planform view, and have a wedge-like section, forming an acute angle between the dorsal and ventral faces of the tool. The width of the working edge must be at least half of the tool’s maximum width. However, it should be noted that while this definition is widely accepted, other definitions for this type do exist (for details see [2]). In light of this strict definition, especially regarding the blank type, cleavers usually appear in assemblages assigned to the large flake Acheulian (LFA) technological tradition. As such, they are found mainly in Acheulian assemblages from Africa, and although they also appear in other parts of the Old World, they have a somewhat more restricted distribution than other components of the Acheulian bifacial toolkit such as the handaxe.

Handaxes have previously received much attention from both amateurs and scholars due to their aesthetic appeal. In recent years handaxes have been a focus of many scholarly attempts to explain their production, use, and their implications for hominin behavior. In contrast, cleavers have received much less attention. In the following paper we provide observations into this tool and analyze them using concepts derived from cognitive neuroscience. This is done to better understand the sophisticated planning behind the production of the cleavers, and the implications this has for the cognitive abilities and behavior of their makers and users.

### Cognitive background

The first step in a cognitive analysis of any prehistoric technology is the identification of attributes that carry cognitive implications, and this selection depends to a large degree on the theoretical grounding of the analysis [3,4]. The current analysis is grounded in the discipline of cognitive neuroscience, which is an interdisciplinary approach that combines the methods and concepts of cognitive psychology with the evidence from neuroscience (neuroimaging and clinical neuropsychology). As this approach has generated an immense literature over the past quarter century, it is necessary to narrow the focus. Here we will rely on two sets of studies: first, neuroimaging research focusing on human and non-human tool use, and second, a cognitive model of expert performance.

Dietrich Stout and colleagues [5–9], and Guy Orban and colleagues [10,11] have used functional magnetic resonance imaging (fMRI) to identify brain activation patterns of humans and macaques using tools. Together these studies have identified a complex pattern of activation that includes the frontal, parietal, and temporal lobes of the brain, and which is centered on the inferior portion of the parietal lobes known as the supra-marginal gyrus. This ‘anthropoid object manipulation network’ (AOMN; Wynn’s terminology) includes circuitry governing visually guided reaching, sequential organization of action, and also mirror neurons that fire when action is observed. Stout’s research has confirmed that the AOMN is the primary system underpinning stone knapping, and he and his colleagues have begun to identify additional neural resources that came into play with more advanced knapping techniques [7].

Describing the neural substrate of tool use is important but it does not capture the richness and subtlety of tool use in natural settings. Cognitive psychology provides an appropriate and well-researched model known as ‘expert performance’ or expertise. This is the kind of thinking that experts use in their particular domains of performance [12,13]. Expert cognition has several salient features: rapid problem assessment, virtually error free execution, ability to shift attention and return to task without loss of information, rapid learning of new routines, and flexible responses to problems that arise during performance. Most famously studied in chess masters, this kind of thinking is clearly the basis of expert tool use as well [14–17]. Expertise relies on long-term memory (LTM), especially a huge body of well-learned procedures. As a novice learns procedures he or she consolidates them into coherent ‘chunks’ of information, and attaches a cue to each chunk, such as a word or an image [13–15]. Hearing, seeing, or accessing the cue will give immediate access to the entire chunk of information. The novice accesses a cue in working memory, which is the amount of information one can hold in attention and process in the presence of interference [18–20]. Expertise is thus a cognitive ability that engages both long-term memory and working memory.

In sum, tool use, including stone knapping, is an expert cognitive system that relies on well-learned long-term procedural memories, and the ability to access them quickly and deploy them appropriately. Expert cognition has definite advantages over the effortful problem solving performed in the active attention of working memory [18]. Because the procedures are well-learned they can be activated immediately through a simple visual or even tactile cue. It is a flexible kind of thinking, but it is not inherently innovative [15]. On the neural level this cognitive system is governed by an object manipulation network that initially evolved with anthropoid primates, but which has been expanded and refined over the course of hominin evolution [8,10].

Here we use the reduction sequence reconstructed for cleavers from the Acheulian site of Gesher Benot Ya‘aqov (GBY) to describe the *chaîne operatoire* used by the knappers. We then use the results of this analysis as the basis for an interpretation of the underlying cognitive strategy.

## Materials and methods

The site of GBY, located in the Northern Jordan Valley, Israel and dated to 780 Ky BP is the only occurrence of *in-situ* LFA assemblages in the Levant [21, 2]. As such it is the only Levantine assemblage that includes cleavers in primary depositional context. We sample here 15 of the archaeological horizons, which together yielded 168 tools (S1 Table). All the excavated material is housed at the Institute of Archaeology at the Hebrew University of Jerusalem.

The meticulous analysis conducted on the GBY lithic assemblages over the years has allowed us to reconstruct details of the *chaîne opératoire* used for biface production. At GBY, this *chaîne opératoire* consisted of several discrete stages, beginning with the procurement of raw materials in the form of basalt slabs from specific outcrops and the acquisition of a series of different sized percussors. The next stage included the fragmentation of the slabs and their reduction into giant cores, which were then modified by the application of several different knapping methods. Subsequently, these cores were used to detach large flakes having a specific set of characteristics and morphology making them appropriate to be used as blanks for the bifaces [22–25]. At GBY, several core reduction methods were identified by the detailed analysis of the giant cores, the finished tools, and a wide range of waste products [25]. These include the Levallois, Kombewa, slab-slicing, bifacial and ad-hoc methods. Selected large flake blanks were then subjected to varying degrees of modification, at times very minimal, which shaped them into finished handaxes and cleavers. The post-detachment modification of the blanks enabled the knappers to mask morphological differences (variations) stemming from differences in the specific production technology, and produce highly uniform assemblages of these tools in terms of their within-group morphological variability. This production sequence, generally applicable to both handaxes and cleavers, provides evidence for planning with regard to the raw material selection, core modification and blank production [22–25].

The strict morphological and technological constraints of the cleavers in comparison to handaxes, reflects their utilitarian function, and required the use of well-established alternative objectives and procedures that provided greater control over the volumetric configuration of the flake. A specific planform and section morphology of the distal working edge was needed to produce a functional and efficient configuration that yielded mass penetration and optimal shearing, making the cleaver an ideal tool for cutting of meat or wood, similar to the modern butcher’s knife. Hence, this particular distal configuration was the main objective of cleaver production and was a requirement for the function of these tools ([26] and references therein). As the working edge cannot be produced by retouch, or other post detachment modifications, its morphology must be dictated by the specific core reduction method prior to the removal of the blank. Hence an alternative procedure was required for the production of cleavers, in contrast to that of handaxes, which were more intensively modified by retouch. The sophistication of this procedure lay in the ability to execute a particular scar pattern on the core’s debitage surface prior to the detachment of the predetermined flake, which in turn formed the particular pre-planned shape of the distal end of the cleaver.

### Renewed analysis of the GBY cleavers

**Fig 1:**
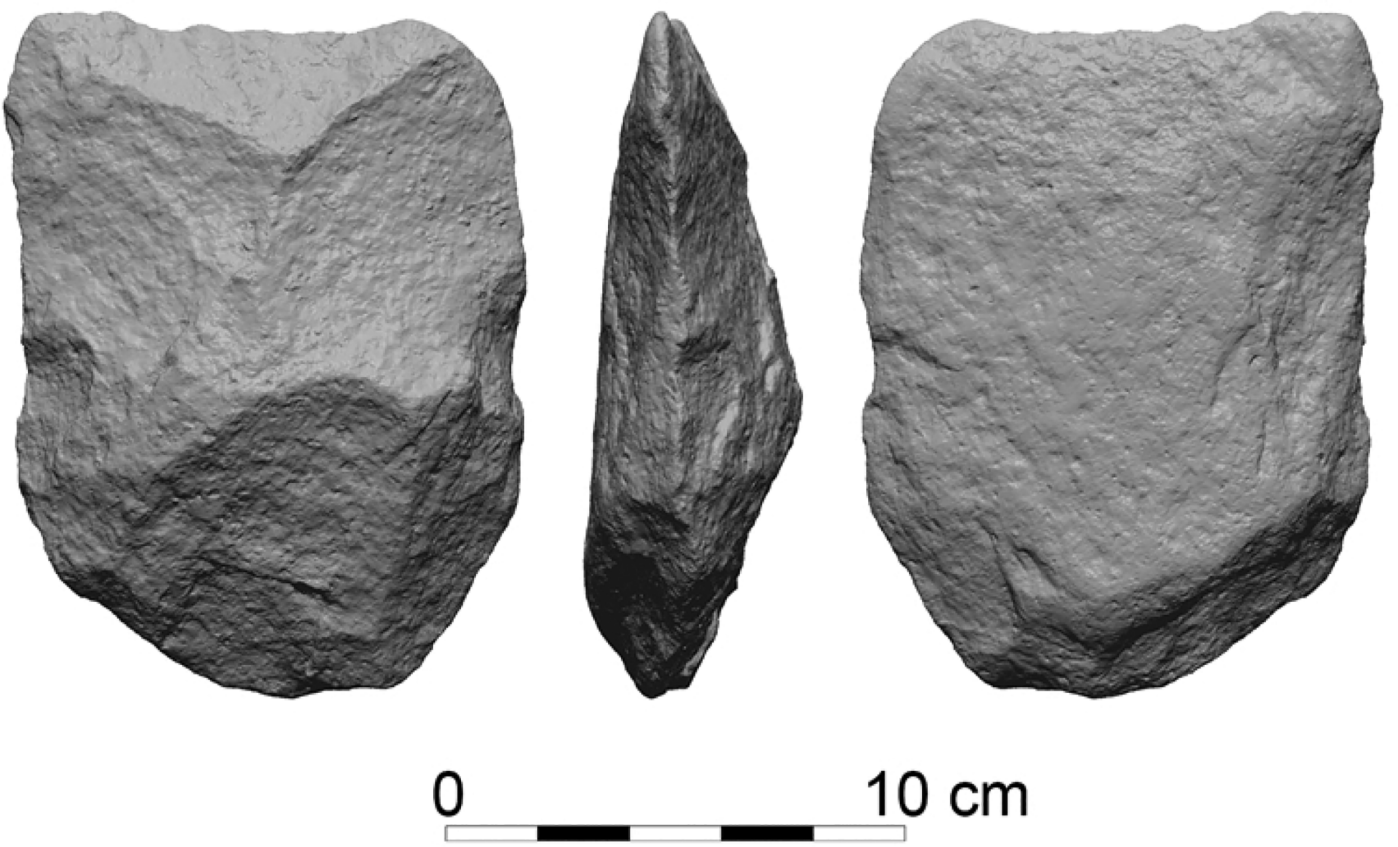
A Basalt cleaver with distal dorsal configuration resulting from a flake scar remnant. GBY#9896, Layer JB.

The detailed analysis of the cleaver component of the GBY assemblages has provided several insights regarding the modes by which the distal morphology and configuration were achieved. There are three different modes for the design of the working edge. The first is the “classical” mode, in which the distal dorsal configuration is achieved by exploiting the scar pattern of the debitage surface of a giant core (Fig 1). The removal of this predetermined flake leaves a remnant of a large negative scar on the distal end of the flake. The intersection between the ventral face and the remnant of the scar on the dorsal face forms the desired working edge morphology. Such production mode is commonly characterized by a slight concavity of the dorsal distal surface forming the working edge, stemming from the morphology of the flake scar.

**Fig 2:**
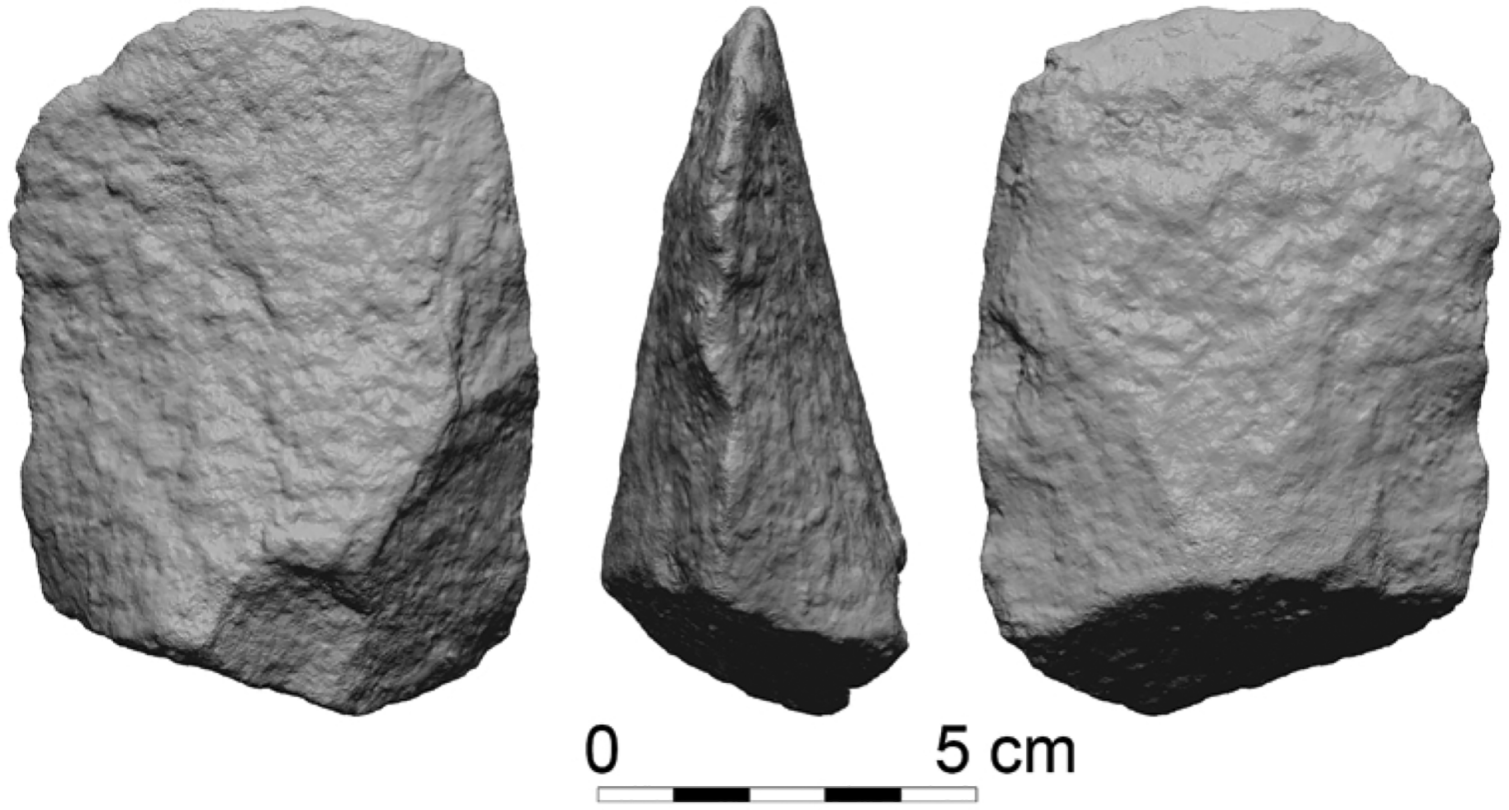
A minimally modified Kombewa basalt cleaver. GBY#100, Layer II-6 Level 4.

The second mode consists of cleavers with an unmodified Kombewa surface as their dorsal face. This, in fact, is a minimally modified, and at times completely unmodified, Kombewa flake (Fig 2). The distal working edge is formed by the intersection of the two surfaces, which are the two ventral surfaces of the Kombewa flake. When post detachment modifications do occur, they are commonly restricted to the lateral edges and proximal end, shaping the striking platform area and thinning the bulb of percussion of the tool, similarly to that of the previous mode.

**Fig 3:**
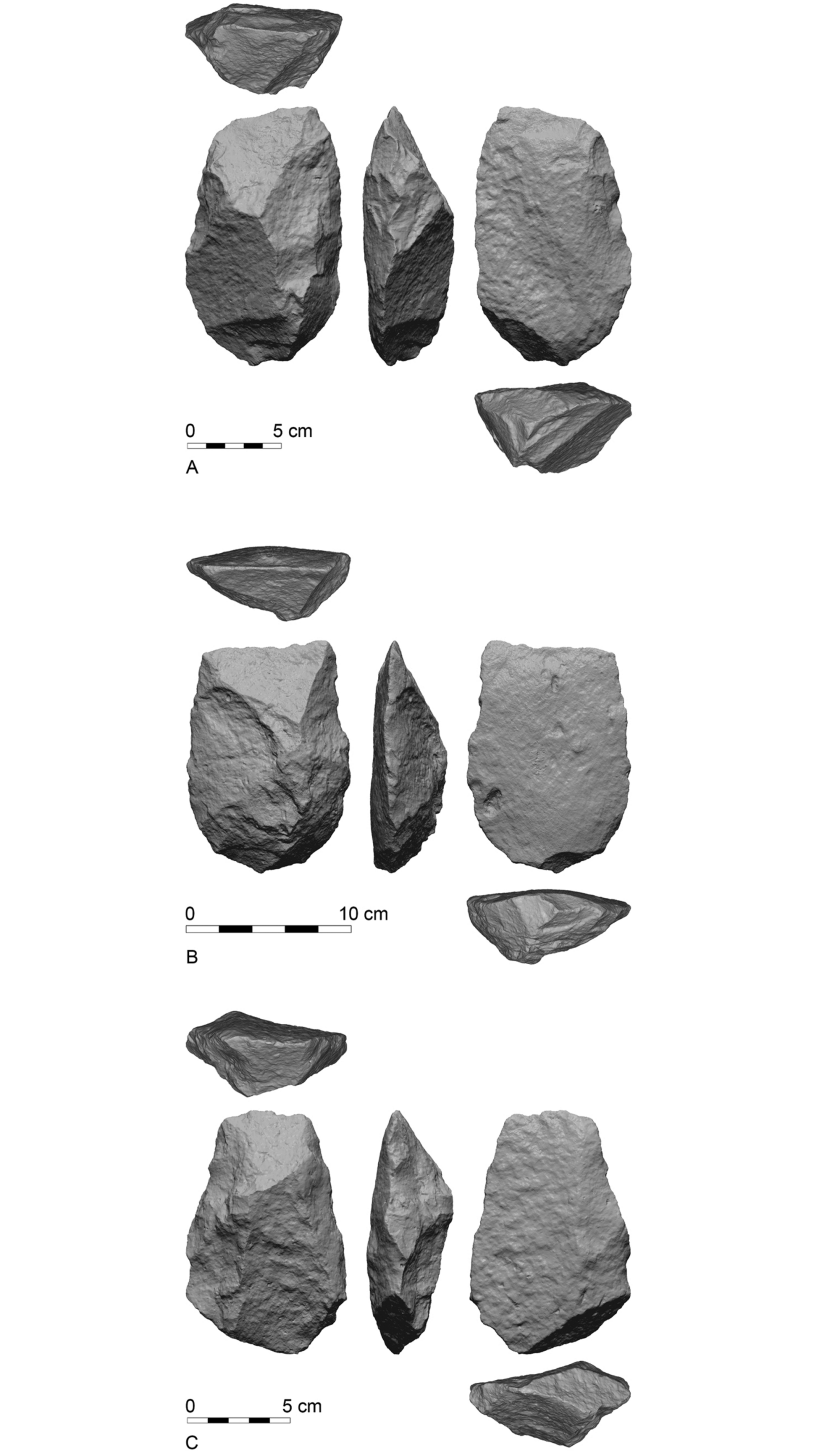
Three basalt cleavers modified by delineation; top: GBY#5906, Layer II-6 Level 3; middle GBY#5976, Layer V-5; bottom: GBY#13765, Layer II-6 Level1.

**Fig 4:**
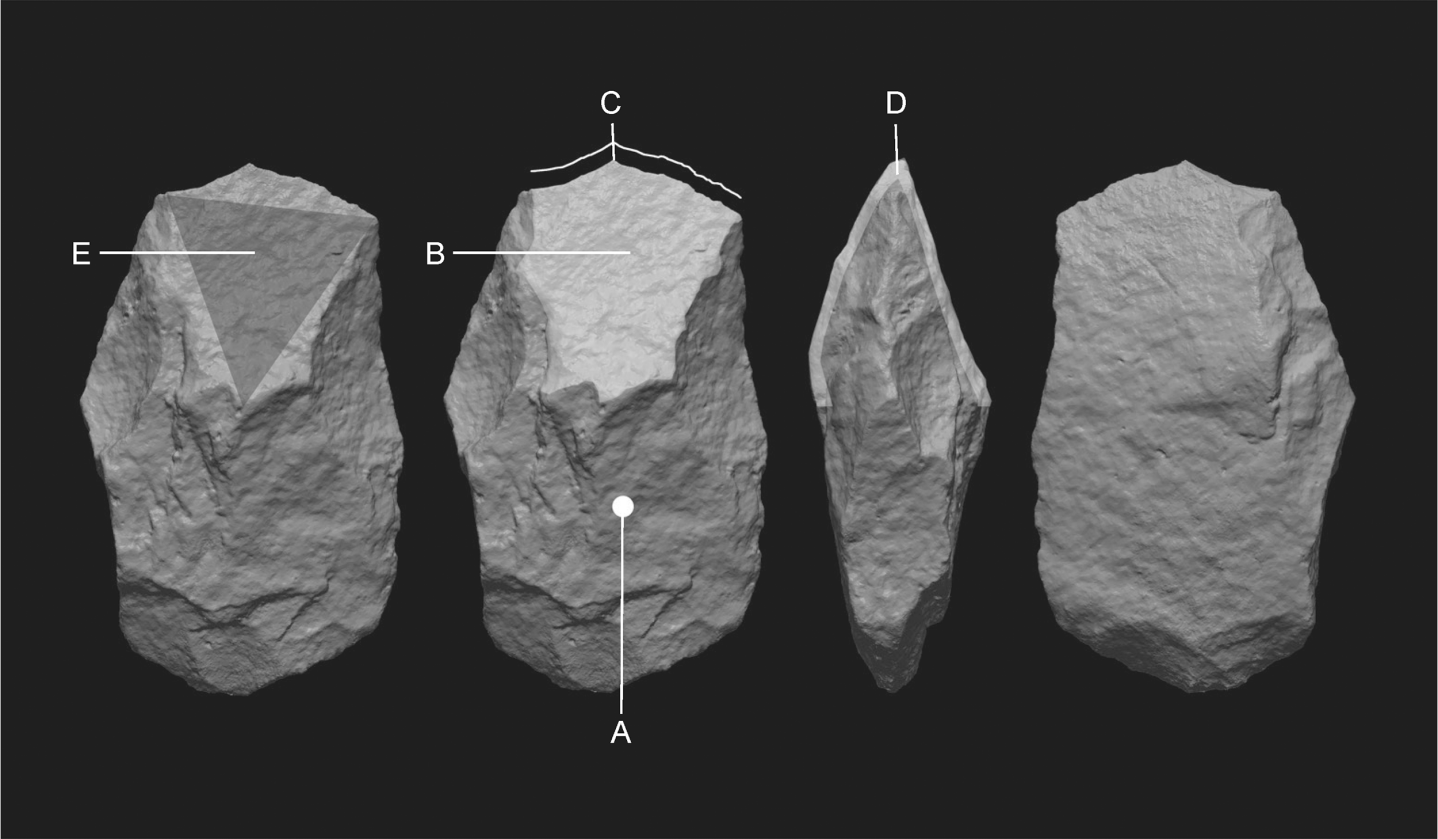
A basalt cleaver modified by delineation (#13763 Layer II-6 Level 3) showing the main features discussed in the text. (A) Invasive retouch covering the majority of the dorsal surface; (B) The flat delineated surface; (C) The working edge; (D) The acute angle formed by the intersection between the delineated dorsal and unmodified ventral faces; (E) The triangular shape formed by the delineated surface.

The third mode consists the post detachment shaping of the blank. In this mode invasive retouch is used to modify the dorsal face of the flake (Figs 3 and 4A). The retouch is performed so that the flake scars will cover most of the surface of the tool, leaving the original surface on the distal end delineated (Fig 4B). This delineated surface is always wither flat or convex, but never concave as in the first mode. The distal edge always remains unmodified (Fig 4C) so that the working edge is formed by the intersection of the delineated dorsal and unmodified ventral surfaces (Fig 4D). In many cases the unretouched delineated surface formed a triangular shape (Fig 4E), in which the base is formed by the straight working edge and its opposing vertex, in the middle part of the cleaver, usually at the thickest point.

In the attempt to gain better insight into the technological characteristics of cleaver production we have examined in detail the modification modes of the GBY cleavers. The recorded observations included the blank type on which the tool was modified (flake, Kombewa and Possibly Kombewa) and the mode by which the cleavers dorsal-distal configuration was attained.

## Results

The following paragraphs present the results of the analyses. In the first sub-section the lithic analysis results will be presented, followed by their behavioral implications. Next, these implications are taken as the basis for a cognitive analysis.

### Lithic analysis

Table 1 presents the frequencies of each dorsal-distal end across the different blank types. The observations for each of the tools are presented in the supporting information (S1 Table).

**Table 1:** The distribution of dorsal distal modification types across the different blank types for the GBY cleavers (excluding indeterminate).

	Dorsal Distal Modification							
	Scar from Core		Surface Delineated by Retouch		Unmodified Surface		Total	
Blank Type	N	%	N	%	N	%	N	%
Flake	50	59.52	32	38.10	2	2.38	84	100.00
Kombewa	0	0.00	5	21.74	18	78.26	23	100.00
Possibly Kombewa	0	0.00	10	55.56	8	44.44	18	100.00
Total	50	40.00	47	37.60	28	22.40	125	100.00

Of the entire studied sample of 168 cleavers, 43 (25.6%) could not be classified to either blank type or modification mode due to continuous weathering. Of the remaining sample, the majority of tools were modified on regular flakes (n = 84, 67.2%). The most common modification mode is that formed by a prior scar on the debitage surface of the core (n = 50, 40%). Naturally, this mode is limited to tools that were modified on regular blanks, as Kombewa (and possibly Kombewa) blanks lack scars of previous removals. The rest of the tools are divided between a group whose distal ends consists of unmodified surface delineated by intensive surface flaking (n = 47, 37.6%) and cleavers lacking any modification of their distal ends (n = 28, 22.4%). Examination of cleavers with unmodified distal ends shows that even though they are produced on all types of blanks, the Kombewa and possibly Kombewa categories consist of almost 93% of the blanks used for this type of distal end configuration. In other words, the probability of having a non-modified distal edge is significantly higher for items produced on Kombewa (or possibly Kombewa) flakes.

Cleavers with distal ends that were modified by delineating a residual surface occur in all types of flakes and in relatively high frequencies. The most common blank of this aspect is a simple flake, followed in descending order by Possibly Kombewa and Kombewa flakes. When considering the origin of the delineated unmodified surface there are two possibilities. The first is that this surface is the original natural flat surface of a basalt slab. The second possibility is that this surface is in fact a remnant of an old ventral face. Our detailed analysis of these items supports the second option due to the slight convexity of this surface on most of the tools in this category. Therefore, the implication of this interpretation is that those blanks classified as simple flakes are in fact Kombewa flakes (or Possibly Kombewa) that were not identified as such due to intensive subsequent modification.

The frequency of Kombewa flakes in the GBY bifacial assemblages has been previously described several times. We have previously reported high values of Kombewa blanks associated with the production of bifaces [21] and lower values in the final report on the lithic assemblages of GBY [25]. We have explained the differences as being the result of two factors: the deterioration and continuous weathering of some of the bifaces on the one hand, and on the other hand a change in our analytical criteria for identifying Kombewa flakes. One criterion that was added over the years is that for an example to be classified as Kombewa flake it had to possess two visible striking platforms. While this criterion rendered the classification of items as Kombewa certain, it also drastically reduced their frequency in the assemblage. Clearly, the results of these analytical differences influenced the frequency of the Possibly Kombewa artifacts. Our current analysis requires only that items present a clear indication of having two ventral faces but not necessarily two striking platforms. Our renewed detailed analysis of the cleavers’ distal ends provides support for the classification of additional items into the Kombewa/Possibly Kombewa categories. If our interpretation is correct, then almost 40% (n = 32) of the cleavers classified as modified on regular flakes were actually modified on flakes produced by the Kombewa technology. This contribution augments the frequency of tools derived of Kombewa technology to a total of 60% (n = 75) of the cleaver assemblage (excluding the indeterminate category). Furthermore, this newly acquired data contributes meaningful new insight to the previously ambiguous nature of the Possible Kombewa category.

### Behavioral implication

With this perspective in hand, it is possible to assess the production procedure of the GBY large-flake cleavers. Several features of the procedure are especially informative:

The procedure consisted of three distinct stages.

- Stage 0—raw material acquisition and percussor acquisition are not tied to the procedures, but are still prerequisites.
- The first stage is the fragmentation of a slab and its transformation to a core.
- The second stage, large flake production, included two alternative modes (at a minimum) for producing flakes with appropriate distal morphology for cleaver production:
  ○ The “classical” method, including Levallois, which exploited scar patterns of the debitage surface of a giant core.
  ○ Unmodified Kombewa flake, with two distinct ventral surfaces.
  ○ Each of these modes included four distinct steps:
    ■ decision to make a cleaver, as opposed to a handaxe,
    ■ preparation of a production surface on a giant core,
    ■ detachment of large flake with an appropriate wedge shaped cross section and cutting edge form,
    ■ assessment: Here the knapper made another choice: if the flake itself had an appropriate morphology, the procedure terminated. If it did not, the knapper went on to a third stage.
- The third stage, when necessary, was guided by three goals:
  ○ Trimming the lateral edges and proximal end to thin the bulb of percussion (or in the case of Kombewa, two bulbs of percussion),
  ○ reduce the volume of the tool,
  ○ in the case of Kombewa flakes, delineate a portion of the original surface on the dorsal-distal part of the flake using invasive retouch

This reconstructed procedure has several provocative behavioral components (i.e., behavioral components that are implied by the procedure):

1. There was a guiding objective. The knappers were not simply producing sharp flakes to be used in a specific task-at-hand. They had a target tool (“cleaver” or “handaxe”) that guided their actions and decisions. The tool itself was the goal. This is an important feature of human technologies. When a chimpanzee has a task to perform he or she selects or modifies a nearby object to complete that task, and when done abandons it [27,28]. Chimpanzees have a task-oriented technology. When we in the modern world want to complete a task, we most often select an appropriate tool, which was not designed for that specific task at hand, but for a set of potential tasks. When we need a new knife, we go to the store (or online) and select a knife. We are concerned with the features of the knife, including its range of potential uses, not shredding today’s cabbage. We have a tool-oriented technology. When a GBY knapper travelled to a quarry, he or she need not have had a specific task-at-hand in mind. Instead, he or she had one or several tools in mind. In light of their different morphologies and production technologies, we assume that handaxes and cleavers differed also in the function they were intended to fulfil, both utilitarian and others. These functional considerations were inherent in the respective handaxe and cleaver concepts. In this sense, GBY bifaces were components of a tool-oriented technology.
2. The procedure included at least two alternative routines for acquiring a blank with appropriate characteristics, one that used the scar pattern of the debitage surface of the core, and one that used the Kombewa method.
3. The procedure included very clear decision points. The first was the choice to make a cleaver. The second concerned the procedure for blank production (classical or Kombewa). After removing the large flake, the knapper made additional choices regarding the modification. In certain cases, only minimal modification consisting of striking platform removal and bulb thinning was applied. In others, the Kombewa surface was further knapped, delineating a residual surface in the dorsal-distal part of the tool. Thus, a knapper’s choice of objective determined the procedure that governed the final outcome. Only in this sense might one conclude that predetermination was in play.

For each alternative there was an established sequence of steps. This was not simply the iterative application of a knapping gesture. One kind of knapping preceded a different kind of knapping in a set sequence.

## Discussion and conclusions

### Archaeological implications

Summing up the different stages of the reduction process of cleavers we are able now to identify several distinct stages concerning the planning capabilities of the GBY hominins. The first is applicable to the production of both handaxes and cleavers and is expressed in the raw material and percussors acquisition phases of the *chaîne opératoire* aimed at biface production. The second refers to the core reduction methods aimed at producing the desired large flakes. Here, however, there is a major difference between handaxes and cleavers. The procedure used in handaxe production allows for greater flexibility late in the process. In contrast, the procedure used in cleaver production requires a decision early in the process about the final configuration of the working edge. This decision then entails a number of specific steps leading to the final product. From the reconstruction of each specific core reduction method at GBY it appears that flakes with appropriate morphologies, dimensions and distal characteristics could only be produced by using the Levallois or the Kombewa methods. This means that the decision to produce a cleaver must have been taken as early as the slab fragmentation and core design stage of the *chaîne opératoire*.

An additional and interesting aspect of our study relates to the dorsal distal modification by delineation of a residual surface. While this appears to be a common modification mode at GBY, it was not a necessary one, as there is a significant number of tools lacking any dorsal distal modification. This observation is even further highlighted given our suggestion that this modification mode was always applied to Kombewa flakes. Clearly, unmodified (or slightly modified) Kombewa flakes provided a similar functionality with regards to the distal morphology that allowed the necessary mass penetration and shearing capacities. Thus, it remains an open question why some Kombewa flakes underwent this additional modification while others did not. We suggest, however, that this selection could be related to stylistic or cultural preferences.

Our results and conclusions shed light and provide additional insights concerning the issue of hominin abilities. It is evident that the GBY hominins, as documented elsewhere in the Acheulian record, were capable of planning, producing and exploiting a wide array of convex surfaces. These abilities are recorded in the production/modification of large predetermined flakes, as well as in the modification of handaxes and cleavers [22–25]. As far as cleavers are concerned, and beyond the general similarities shared with handaxes, controlling the convex surfaces through delineation by retouch was yet another central concept in cleaver modifications.

The exploitation of convex surfaces, so exceptionally well controlled in the Acheulian, is documented in much earlier times (ca. 1.6 Ma) particularly in the production of cores on flakes reported from the Acheulian site of ‘Ubeidiya [29]. The same phenomenon of cores on flakes is well represented in the flint assemblages of GBY and in abundance [25]. A different facet of this mode of exploitation occurs in many Acheulian assemblages where bifaces were transformed into cores by a removal of a large predetermined flake from the convex surface of bifaces (e.g. [30–32]).

The ability to manipulate the convex surfaces independently of the artifacts size (bifaces, large flakes and very small ones) is a remarkable trait of the Acheulian. Furthermore, it is the foundation of many of the Middle Paleolithic cultural entities, and in particularly those associated with the Levallois method, as exploitation of convex surfaces is a fundamental prerequisite of this system. The cognitive abilities that enable designing a core in order to obtain a morphologically predetermined flake, and to exploit and modify convex surfaces form the roots of the evolution of the Levallois method [25].

### Cognitive implications

What, then, are the cognitive implications of these behavioral patterns? Falling back on the theoretical framework summarized briefly earlier, and comparing the GBY knappers to earlier tool users, we suggest the following:

A The GBY knappers possessed a clear tool concept. This is an underappreciated ability that arose with the first bifaces [33,34]. As described above, non-human primate tool use is task-oriented, not tool-oriented. This is arguably also true of the earliest lithic technology, the Oldowan [35]. The task-at-hand was to cut meat or to extract termites, to cite two examples. The tools were components of that task, but not the goal. The cleavers at GBY were the end goal; early in the procedure the knapper made choices that dictated a final product. The cleavers could then, of course, be used, but no specific use guided the procedure; instead, a tool model guided the procedure
B The GBY knappers also possessed a cleaver concept that was distinct from the handaxe concept. The GBY cleaver production procedure differed from the one used for handaxes. The knappers chose, at the outset, to make cleavers. Thus, the tool concept subsumed at least two sub-categories, and we suspect several more (each with distinct procedures). These procedures would have been held in long-term memory (LTM), and if expert cognition is a reliable guide, each would have had a semantic tag (gesture or word) that could act as a cue [13–15].

The precise cognitive nature of these concepts is far from clear. ‘Tool’ need not have been a ‘mental template’ in the sense of a stable visual representation imagined ahead of time; indeed, given that the category ‘tool’ clearly subsumed several specific tool types (e.g., cleaver), it could not have been a simple visual target. ‘Tool’ is an abstract category for us–it is a category made up of categories—and must have been for the GBY knappers as well. ‘Cleaver’, on the other hand was a more concrete category that almost certainly did have specific visual and ergonomic components. It existed as a model against which each large flake was evaluated. When modern humans use named tools (e.g., screwdriver) areas of the fusiform gyrus are activated [10] that are not activated when non-human primates use tools. Thus, thinking in terms of tool categories appears to have evolved at some point in hominin evolution. We suspect that this evolutionary development occurred long before the time of GBY, but it is certainly in evidence at GBY. Moreover, ‘tool’ and ‘cleaver’ were shared concepts that must have been acquired through social learning [36,37].

This evidence for cognitive categories (‘tool’ ‘cleaver’ ‘handaxe’) is, we think, very important. Anthropoid technical cognition evolved initially to control sequential procedures, which remain critical components of technical expertise. Categorical knowledge, however, is not a necessary component of anthropoid technical cognition; there is no reason to suppose that chimpanzees possess an abstract category of ‘tool’, or even a concrete concept ‘termite probe.’ Modern technical expertise is replete with categories that are held in LTM in the form of declarative/semantic information. Procedural LTMs are still paramount, but modern experts also rely on well-learned categories. It would appear that this was true for the GBY knappers, as well. The number of categories may have been fewer than one would find with a modern expert, but categories were definitely present in the minds of the GBY knappers. Further, it would seem likely, though this is impossible to know, that the GBY knappers had declarative/semantic labels for these concepts, either in the form of vocal words or perhaps gestures (we favor the former).

C GBY lithic technology was governed by expert cognition. The nested organization of stages and discrete steps is precisely the kind of organization that underpins modern craft production [14]. It relied on the neural resources of long-term procedural memory, the construction of retrieval structures linked to cues, and the resources of working memory (WM) to hold these retrieval structures in attention. From an evolutionary perspective, expertise evolved through the expansion of both LTM capacity and working memory capacity. Compared to non-human primate tool use and earlier Oldowan lithic technology, GBY cleaver production required more and longer procedural sequences held in LTM, and also an increase in working memory capacity [38,39]. WM is the amount of information one can hold in attention *and* process in the presence of interference. Adding a cleaver concept to the general motor procedure would alone have required the knappers to consider the tool-as-end-goal while they knapped. It would also have required more active monitoring of the knapping process itself, as would the distinct decisions evident at three points in the reduction sequence. Inattentive, iterative knapping could not have produced these artifacts. The third stage of the cleaver production procedure–additional trimming–also implicates an increase in WM capacity. The knappers had three objectives: thinning the bulb of percussion, mass reduction and delineation of a smooth surface at the dorsal distal part of the tool. The knapper had to shift attention from one to other while knapping, and this attention shift is a component of the ‘cognitive control’ resources of working memory [18]. Cognitive control includes other monitoring abilities, including response inhibition (suppressing an automatic response when necessary) and maintenance of sequence order. The cleaver production procedure was not a simple iterative series, but a sequence that included very specific shifts in short term goals that were embedded within the larger goal, and thus required cognitive control. The GBY cleaver procedures confirm that the hominins living at GBY used the same cognitive control resources that are evident in modern expert craft production. Subsequent to GBY expert cognition undoubtedly continued to evolve via enhancements in LTM and WM, but the basic pattern of expert technical cognition was clearly in place 780,000 years ago.

In sum, when compared to earlier stone knapping procedures, and the tool-use of non-human primates, the GBY knappers demonstrated significant cognitive developments. Most salient are the clear reliance on cognitive categories, which were socially learned, and significant expansions of long-term memory capacity and working memory capacity. It also appears likely to us that the knappers possessed semantic labels for their concepts. Compared to modern technical expertise, such blacksmithing [14] the GBY knappers demonstrated fewer cognitive categories. However, the archaeological record preserves only a very narrow range of technical activities, especially for the time depth of GBY, and a comparison with modern craft production is almost certainly misleading. What is more significant is the evidence for hominin reliance 780,000 years ago on a cognitive strategy—expert cognition—that continues to be important in the modern world.

## Supporting information

S1 Table Blank and dorsal-distal modification type for each of the artifacts included in the analysis. (DOCX)

## References

[1] Tixier J. Le hachereau dans l'Acheuléen Nord-Africain: Notes typologiques. In: Patte E, editor. Congrès préhistorique de France, XVème session. Paris: Sociètè Prèhistorique Française; 1957. pp. 914–923.

[2] Sharon G. Acheulian large flake industries: Technology, chronology, and significance. Oxford: BAR International Series; 2007.

[3] Botha R. Prehistoric shell beads as a window on language evolution. Language & Communication. 2008;28: 197–212.

[4] Wynn T. Hafted spears and the archaeology of mind. Proceedings of the National Academy of Sciences. 2009;106: 9544–9545. 10.1073/pnas.0904369106 PMC2701010 19506246

[5] Hecht EE, Gutman DA, Bradley BA, Preuss TM, Stout D. Virtual dissection and comparative connectivity of the superior longitudinal fasciculus in chimpanzees and humans. Neuroimage. 2015;108: 124–137. doi: 10.1016/j.neuroimage.2014.12.039 25534109 10.1016/j.neuroimage.2014.12.039 PMC4324003

[6] Hecht EE, Gutman DA, Khreisheh N, Taylor SV, Kilner J, Faisal AA, et al Acquisition of Paleolithic toolmaking abilities involves structural remodeling to inferior frontoparietal regions. Brain Structure and Function. 2015;220: 2315–2331 doi: 10.1007/s00429-014-0789-6 24859884 10.1007/s00429-014-0789-6

[7] Stout D, Apel J, Commander J, Roberts M. Late Acheulean technology and cognition at Boxgrove, UK. Journal of Archaeological Science. 2014;41: 576–590.

[8] Stout D, Hecht E, Khreisheh N, Bradley B, Chaminade T. Cognitive demands of Lower Paleolithic toolmaking. PLoS One. 2015;10: e0121804 doi: 10.1371/journal.pone.0121804 25875283 10.1371/journal.pone.0121804 PMC4398452

[9] Stout D, Passingham R, Frith C, Apel J, Chaminade T. Technology, expertise and social cognition in human evolution. European Journal of Neuroscience. 2011;33: 1328–1338. doi: 10.1111/j.1460-9568.2011.07619.x 21375598 10.1111/j.1460-9568.2011.07619.x

[10] Orban GA, Caruana F. The neural basis of human tool use. Frontiers in psychology. 2014;5. 10.3389/fpsyg.2014.00310 PMC3988392 24782809

[11] Orban GA, Claeys K, Nelissen K, Smans R, Sunaert S, Todd JT, et al Mapping the parietal cortex of human and non-human primates. Neuropsychologia. 2006;44: 2647–2667. doi: 10.1016/j.neuropsychologia.2005.11.001 16343560 10.1016/j.neuropsychologia.2005.11.001

[12] Gobet F. Expert memory: A comparison of four theories. Cognition. 1998;66: 115–152. 9677761 10.1016/s0010-0277(98)00020-1

[13] Gobet F, Simon HA. Five seconds or sixty? Presentation time in expert memory. Cognitive Science. 2000;24: 651–682.

[14] Keller CM, Keller JD. Cognition and tool use: The blacksmith at work. New York: Cambridge University Press; 1996.

[15] Wynn T, Coolidge FL. Technical cognition, working memory and creativity. Pragmatics & Cognition. 2014;22: 45–63.

[16] Wynn T, Coolidge FL. The expert Neandertal mind. Journal of human evolution. 2004;46: 467–487. doi: 10.1016/j.jhevol.2004.01.005 15066380 10.1016/j.jhevol.2004.01.005

[17] Wynn T, Coolidge FL. How Levallois reduction is similar to, and not similar to, playing chess In: Nowell A, Davidson I, editors. Stone tools and the evolution of human cognition. Boulder: University Press of Colorado; 2010 pp. 83–103.

[18] Baddeley A. Working memory, thought, and action. Oxford: Oxford University Press; 2007.

[19] Ericsson KA, Delaney PF. Long-term working memory as an alternative to capacity models of working memory in everyday skilled performance In: Miyake A, Shah P, editors. Models of working memory: Mechanisms of active maintenance and executive control. New York: Cambridge University Press; 1999 pp. 257–97.

[20] Ericsson KA, Kintsch W. Long-term working memory. Psychological review. 1995;102: 211–245. 7740089 10.1037/0033-295x.102.2.211

[21] Goren-Inbar N, Saragusti I. An Acheulian biface assemblage from the site of Gesher Benot Ya'aqov, Israel: Indications of African affinities. Journal of Field Archaeology. 1996;23: 15–30.

[22] Madsen B, Goren-Inbar N. Acheulian giant core technology and beyond: An archaeological and experimental case study. Eurasian Prehistory. 2004;2: 3–52.

[23] Goren-Inbar N. Culture and cognition in the Acheulian industry—A case study from Gesher Benot Ya'aqov. Philosophical Transactions of the Royal Society B. 2011;366: 1038–1049 10.1098/rstb.2010.0365 PMC3049101 21357226

[24] Goren-Inbar N, Grosman L, Sharon G. The technology and significance of the Acheulian giant cores of Gesher Benot Ya'aqov, Israel. Journal of Archaeological Science. 2011;38: 1901–1917.

[25] Goren Inbar N, Alperson-Afil N, Sharon G, Herzlinger G. The Acheulian site of Gesher Benot Ya‘aqov: The Lithic Assemblages. Dordrecht: Springer; forthcoming.

[26] Mourre V. Implications culturelles de la technologie des hachereaux. Ph.D. Thesis, Paris 10; 2003.

[27] Goodall J. The chimpanzees of Gombe: Patterns of behavior. Cambridge: Belknap Press; 1986.

[28] McGrew WC. Chimpanzee material culture: Implications for human evolution. Cambridge: Cambridge University Press; 1992.

[29] Bar-Yosef O, Goren-Inbar N. The lithic assemblages of ‘Ubeidiya. Jerusalem: Institute of Archaeology, Hebrew University; 1993.

[30] deBono H, Goren-Inbar N. Note on a link between Acheulian and Levallois technologies. Mitekufat Ha'even. 2001;31: 9–23.

[31] Matskevich Z, Goren-Inbar N, Gaudzinski S. A newly identified Acheulian handaxe type at Tabun Cave: The Faustkeilblätter In: Cook J, editor. A very remote period indeed: Papers on the Palaeolithic presented to Derek Roe. Oxford: Oxbow Books; 2001 pp. 120–132.

[32] Marder O, Milevski I, Matskevich Z. The handaxes of Revadim Quarry: Typo-technological considerations and aspects of intra-site variability In: Goren-Inbar N, Sharon G, editors. Axe age: Acheulian tool-making from quarry to discard. London: Equinox; 2006 pp. 223–42.

[33] Wynn, T. & Berlant, T. The handaxe aesthetic. In: Coolidge FC, Overmann K, editors. Squeezing minds from stones. Oxford: Oxford University Press; forthcoming.

[34] Gowlett J. The elements of design form in Acheulian bifaces: Modes, modalities, rules and language In: Goren-Inbar N, Sharon G, editors. Axe age: Acheulian toolmaking from quarry to discard. London: Equinox; 2006 pp. 203–22.

[35] Wynn T, Hernandez‐Aguilar RA, Marchant LF, Mcgrew WC. “An ape's view of the Oldowan” revisited. Evolutionary Anthropology: Issues, News, and Reviews. 2011;20: 181–197. 10.1002/evan.20323 22034236

[36] Cole J. Accessing hominin cognition: Language and social signaling in the Lower to Middle Palaeolithic In: Wynn T, Coolidge FL, editors. Cognitive models in Palaeolithic archaeology. Oxford: Oxford University Press; 2017 pp. 157–96.

[37] Shipton C. Imitation and shared intentionality in the Acheulean. Cambridge Archaeological Journal 2010:20: 197–210.

[38] Coolidge FL, Wynn T. The rise of Homo Sapiens: The evolution of modern thinking. Chichester: John Wiley & Sons; 2009.

[39] Gibson KR. New perspectives on instincts and intelligence: Brain size and the emergence of hierarchical mental constructional skills In: Parker ST, Gibson KR, editors. “Language" and intelligence in monkeys and apes: Comparative developmental perspectives. New York: Cambridge University Press; 1990 pp. 97–128.

