# Supplementary material for: Expert cognition in the production sequence of Acheulian cleavers at Gesher Benot Ya'aqov, Israel: A lithic and cognitive analysis
Source: PLoS One. 2017 Nov 16;12(11):e0188337. doi: 10.1371/journal.pone.0188337 (PMC5690685; doi:10.1371/journal.pone.0188337)
Supplement: S1 Table — (DOCX) [file pone.0188337.s001.docx]

Table S1: Blank and dorsal-distal modification type for each of the artifacts included in the analysis

| No. | Layer | Blank | Dorsal-Distal Modification |
| --- | --- | --- | --- |
| 13765 | II-6/L1 | Flake | Scar of Core |
| 5448 | II-6/L1 | Flake | Scar of Core |
| 5653 | II-6/L1 | Flake | Indeterminate |
| 5291 | II-6/L1 | Kombewa | Unmodified Kombewa Surface |
| 2121 | II-6/L1 | Kombewa | Unmodified Kombewa Surface |
| 5659 | II-6/L1 | Possibly Kombewa | Scar Delineated by Knapping |
| 995 | II-6/L1 | Possibly Kombewa | Scar Delineated by Knapping |
| 5670 | II-6/L1 | Flake | Scar Delineated by Knapping |
| 5614 | II-6/L1 | Kombewa | Scar Delineated by Knapping |
| 5617 | II-6/L1 | Flake | Scar of Core |
| 5636 | II-6/L1 | Flake | Indeterminate |
| 2130 | II-6/L1 | Flake | Scar Delineated by Knapping |
| 5252 | II-6/L1 | Flake | Indeterminate |
| 14196 | II-6/L2 | Flake | Scar Delineated by Knapping |
| 14185 | II-6/L2 | Flake | Scar Delineated by Knapping |
| 14188 | II-6/L2 | Flake | Indeterminate |
| 13761 | II-6/L2 | Flake | Scar of Core |
| 8638 | II-6/L2 | Flake | Scar Delineated by Knapping |
| 8657 | II-6/L2 | Flake | Indeterminate |
| 5674 | II-6/L2 | Flake | Scar of Core |
| 5663 | II-6/L2 | Flake | Scar Delineated by Knapping |
| 5656 | II-6/L2 | Flake | Scar of Core |
| 13757 | II-6/L2 | Flake | Scar Delineated by Knapping |
| 13763 | II-6/L3 | Possibly Kombewa | Scar Delineated by Knapping |
| 13762 | II-6/L3 | Flake | Indeterminate |
| 8664 | II-6/L3 | Kombewa | Indeterminate |
| 8673 | II-6/L3 | Flake | Indeterminate |
| 8569 | II-6/L3 | Possibly Kombewa | Scar Delineated by Knapping |
| 8666 | II-6/L3 | Possibly Kombewa | Scar Delineated by Knapping |
| 8646 | II-6/L3 | Flake | Scar Delineated by Knapping |
| 8654 | II-6/L3 | Possibly Kombewa | Indeterminate |
| 8665 | II-6/L3 | Possibly Kombewa | Indeterminate |
| 5906 | II-6/L3 | Flake | Scar Delineated by Knapping |
| 5908 | II-6/L3 | Flake | Indeterminate |
| 8588 | II-6/L3 | Kombewa | Unmodified Kombewa Surface |
| 1013 | II-6/L4 | Possibly Kombewa | Unmodified Kombewa Surface |
| 1029 | II-6/L4 | Possibly Kombewa | Indeterminate |
| 5130 | II-6/L4 | Flake | Indeterminate |
| 5131 | II-6/L4 | Flake | Scar Delineated by Knapping |
| 5001 | II-6/L4 | Possibly Kombewa | Scar Delineated by Knapping |
| 4996 | II-6/L4 | Flake | Scar of Core |
| 5003 | II-6/L4 | Flake | Scar of Core |
| 5007 | II-6/L4 | Kombewa | Scar Delineated by Knapping |
| 5022 | II-6/L4 | Flake | Indeterminate |
| 5023 | II-6/L4 | Possibly Kombewa | Unmodified Kombewa Surface |
| 5024 | II-6/L4 | Kombewa | Unmodified Kombewa Surface |
| 5025 | II-6/L4 | Kombewa | Unmodified Kombewa Surface |
| 5026 | II-6/L4 | Possibly Kombewa | Indeterminate |
| 5829 | II-6/L4 | Flake | Indeterminate |
| 1056 | II-6/L4 | Flake | Scar Delineated by Knapping |
| 204 | II-6/L4 | Flake | Scar Delineated by Knapping |
| 165 | II-6/L4 | Possibly Kombewa | Scar Delineated by Knapping |
| 33 | II-6/L4 | Flake | Scar of Core |
| 238 | II-6/L4 | Flake | Scar Delineated by Knapping |
| 121 | II-6/L4 | Kombewa | Scar Delineated by Knapping |
| 199 | II-6/L4 | Kombewa | Indeterminate |
| 167 | II-6/L4 | Flake | Indeterminate |
| 184 | II-6/L4 | Kombewa | Unmodified Kombewa Surface |
| 164 | II-6/L4 | Kombewa | Unmodified Kombewa Surface |
| 182 | II-6/L4 | Kombewa | Unmodified Kombewa Surface |
| 186 | II-6/L4 | Kombewa | Unmodified Kombewa Surface |
| 4 | II-6/L4 | Possibly Kombewa | Unmodified Kombewa Surface |
| 90 | II-6/L4 | Kombewa | Unmodified Kombewa Surface |
| 93 | II-6/L4 | Flake | Scar Delineated by Knapping |
| 100 | II-6/L4 | Kombewa | Unmodified Kombewa Surface |
| 180 | II-6/L4 | Flake | Scar Delineated by Knapping |
| 151 | II-6/L4 | Possibly Kombewa | Unmodified Kombewa Surface |
| 296 | II-6/L4 | Flake | Scar Delineated by Knapping |
| 17122 | II-6/L4 | Flake | Scar Delineated by Knapping |
| 292 | II-6/L4 | Flake | Scar of Core |
| 142 | II-6/L4 | Flake | Scar of Core |
| 5008 | II-6/L4 | Flake | Scar of Core |
| 5128 | II-6/L4 | Indeterminate | Indeterminate |
| 5129 | II-6/L4 | Flake | Scar of Core |
| 336 | II-6/L4 | Flake | Scar of Core |
| 333 | II-6/L4 | Flake | Indeterminate |
| 331 | II-6/L4 | Kombewa | Unmodified Kombewa Surface |
| 332 | II-6/L4 | Kombewa | Unmodified Kombewa Surface |
| 345 | II-6/L4 | Flake | Indeterminate |
| 435 | II-6/L4 | Flake | Scar Delineated by Knapping |
| 453 | II-6/L4 | Flake | Scar of Core |
| 449 | II-6/L4 | Flake | Scar of Core |
| 452 | II-6/L4 | Kombewa | Unmodified Kombewa Surface |
| 212 | II-6/L4 | Flake | Scar Delineated by Knapping |
| 223 | II-6/L4 | Kombewa | Scar Delineated by Knapping |
| 220 | II-6/L4 | Possibly Kombewa | Scar Delineated by Knapping |
| 213 | II-6/L4 | Flake | Scar of Core |
| 248 | II-6/L4 | Flake | Scar of Core |
| 226 | II-6/L4 | Flake | Indeterminate |
| 66 | II-6/L4 | Kombewa | Unmodified Kombewa Surface |
| 70 | II-6/L4 | Flake | Indeterminate |
| 269 | II-6/L4 | Flake | Scar Delineated by Knapping |
| 112 | II-6/L4 | Flake | Scar Delineated by Knapping |
| 5133 | II-6/L4 | Flake | Scar of Core |
| 5714 | II-6/L4b | Flake | Scar Delineated by Knapping |
| 5761 | II-6/L4b | Possibly Kombewa | Scar Delineated by Knapping |
| 5777 | II-6/L4b | Flake | Scar of Core |
| 5779 | II-6/L4b | Flake | Scar Delineated by Knapping |
| 5780 | II-6/L4b | Flake | Scar of Core |
| 5778 | II-6/L4b | Flake | Indeterminate |
| 5781 | II-6/L4b | Flake | Scar of Core |
| 5784 | II-6/L4b | Flake | Scar of Core |
| 5862 | II-6/L4b | Flake | Indeterminate |
| 7741 | II-6/L4b | Flake | Scar of Core |
| 1102 | II-6/L4b | Possibly Kombewa | Unmodified Kombewa Surface |
| 1097 | II-6/L4b | Flake | Scar Delineated by Knapping |
| 1089 | II-6/L4b | Flake | Indeterminate |
| 1046 | II-6/L4b | Flake | Indeterminate |
| 1098 | II-6/L4b | Kombewa | Unmodified Kombewa Surface |
| 1058 | II-6/L4b | Possibly Kombewa | Unmodified Kombewa Surface |
| 1042 | II-6/L4b | Flake | Indeterminate |
| 6144 | II-6/L4b | Flake | Unmodified Kombewa Surface |
| 11961 | II-6/L5 | Possibly Kombewa | Unmodified Kombewa Surface |
| 5794 | II-6/L5 | Flake | Scar of Core |
| 5765 | II-6/L5 | Flake | Indeterminate |
| 5835 | II-6/L5 | Flake | Scar Delineated by Knapping |
| 8156 | II-6/L5 | Kombewa | Unmodified Kombewa Surface |
| 9155 | II-6/L5 | Indeterminate | Indeterminate |
| 7061 | II-6/L5 | Flake | Unmodified Kombewa Surface |
| 5793 | II-6/L6 | Flake | Scar Delineated by Knapping |
| 7754 | II-6/L6 | Flake | Indeterminate |
| 7752 | II-6/L6 | Flake | Indeterminate |
| 7744 | II-6/L6 | Flake | Indeterminate |
| 7715 | II-6/L6 | Flake | Indeterminate |
| 7755 | II-6/L6 | Flake | Scar Delineated by Knapping |
| 11963 | II-6/L6 | Flake | Scar of Core |
| 7717 | II-6/L6 | Kombewa | Scar Delineated by Knapping |
| 5792 | II-6/L6 | Flake | Scar of Core |
| 7760 | II-6/L6 | Flake | Indeterminate |
| 5797 | II-6/L6 | Flake | Scar Delineated by Knapping |
| 7738 | II-6/L7 | Flake | Scar Delineated by Knapping |
| 7730 | II-6/L7 | Flake | Scar of Core |
| 7739 | II-6/L7 | Kombewa | Unmodified Kombewa Surface |
| 7735 | II-6/L7 | Flake | Scar of Core |
| 7737 | II-6/L7 | Flake | Scar of Core |
| 7783 | II-6/L7 | Flake | Scar of Core |
| 7784 | II-6/L7 | Flake | Scar Delineated by Knapping |
| 7764 | II-6/L7 | Flake | Scar Delineated by Knapping |
| 7765 | II-6/L7 | Flake | Indeterminate |
| 7769 | II-6/L7 | Flake | Indeterminate |
| 13771 | II-6/L7 | Flake | Scar of Core |
| 14203 | II-6/L7 | Flake | Indeterminate |
| 5839 | JB | Flake | Scar of Core |
| 9898 | JB | Flake | Scar of Core |
| 7789 | JB | Flake | Scar of Core |
| 9896 | JB | Flake | Scar of Core |
| 9897 | JB | Flake | Scar of Core |
| 176 | JB | Flake | Scar of Core |
| 5227 | Unconf-B | Flake | Scar of Core |
| 16594 | Unconf-B | Possibly Kombewa | Unmodified Kombewa Surface |
| 8244 | V-3/4 | Flake | Scar of Core |
| 8255 | V-3/4 | Flake | Scar of Core |
| 8249 | V-3/4 | Possibly Kombewa | Scar Delineated by Knapping |
| 8248 | V-3/4 | Possibly Kombewa | Indeterminate |
| 8243 | V-3/4 | Flake | Indeterminate |
| 8250 | V-3/4 | Flake | Scar of Core |
| 8253 | V-3/4 | Flake | Scar of Core |
| 8247 | V-3/4 | Flake | Scar Delineated by Knapping |
| 5976 | V-5 | Flake | Scar Delineated by Knapping |
| 5904 | V-5 | Flake | Scar of Core |
| 5974 | V-6 | Flake | Scar of Core |
| 5975 | V-6 | Flake | Scar of Core |
| 5977 | V-6 | Kombewa | Indeterminate |
| 8260 | V-6 | Flake | Scar Delineated by Knapping |
| 7528 | V-6 | Flake | Scar of Core |
| 2008 | II-5 | Flake | Indeterminate |
| 2127 | II-5 | Flake | Indeterminate |
| 2136 | II-5/6 | Flake | Scar of Core |
